# Are Lucid Dreams Good for Us? Are We Asking the Right Question? A Call for Caution in Lucid Dream Research

**DOI:** 10.3389/fnins.2019.01423

**Published:** 2020-01-24

**Authors:** Nirit Soffer-Dudek

**Affiliations:** Department of Psychology, Ben-Gurion University of the Negev, Beer-Sheva, Israel

**Keywords:** lucid dreams, induction, nightmares, lucid dreaming therapy, sleep quality, sleep hygiene, dissociation, schizotypy

Lucid dreams (LD), i.e., dreams in which one is cognizant of the fact that one is dreaming, have become well-known in recent years, and their deliberate induction has become widespread. This popularity partly stems from theoretical notions conceptualizing LD as adaptive to mental health. However, empirical evidence for that is equivocal. Moreover, there are at least two reasons why frequent deliberate LD induction may theoretically also be hypothesized to be deleterious to mental health: (1) possibly disrupted sleep quality and (2) possibly disrupted reality–fantasy boundaries. Below, I will discuss evidence regarding relations of LD with well-being vs. psychopathology and then consider each of these two potential disruptions. I will conclude by suggesting that the focus on potential benefits of LD is accompanied by a disregard of potential risks of frequent deliberate LD induction.

LD have become increasingly well-known in recent years, with representations in popular media (e.g., the 2010 sci-fi thriller “Inception”) and several cyber-forums and blogs dedicated to the topic, with thousands of participants (e.g., the Dreamviews LD forum^1^ has over 93,000 members). LD may occur spontaneously, yet techniques for deliberate LD induction (e.g., LaBerge and Rheingold, 1990) have been gaining popularity. Induction techniques have become so popular that 35% of first-year Psychology undergraduate students had tried to deliberately initiate LD at least once (Aviram and Soffer-Dudek, 2018).

Why do people wish to volitionally induce LD? Partly because it is tempting to enter—without using any physical substance—a state of altered consciousness in which one can perform feats not possible in real life (e.g., flying), by exerting control over the dream scenario. But also, since LD are considered by many as an ideal state, promoting well-being and psychological growth (Tholey, 1988; Green and McCreery, 1994), a stance readily adopted by bloggers. Reasons for lucid dreaming are usually wish fulfillment and problem-solving, although many also report aims such as overcoming fears and healing (Stumbrys and Erlacher, 2016). However, as will be reviewed below, empirical evidence in favor of LD as promoting psychological well-being is equivocal, and also, despite common notions, empirical evidence suggests that most LD are not characterized by an ability to control dream events.

LD are considered as indicating mental health and well-being (Snyder and Gackenbach, 1988; LaBerge, 2014). Indeed, in one study they were associated with increased mental health and self-confidence (Doll et al., 2009). Another study exploring LD and personality found that lucid dreamers were socially bold, dominant, experimenting, enthusiastic, and warm (Gruber et al., 1995). LD have also been associated with creativity (Blagrove and Hartnell, 2000) and with psychological resilience in the face of traumatic stress (Soffer-Dudek et al., 2011). Although LD may often be triggered by nightmares, they tend to conclude with positive emotion (Aviram and Soffer-Dudek, 2018). According to Dresler et al. (2015), neurocognitive evidence suggests that insight into dreaming (LD) may be a model for insight into one's illness in schizophrenia, which is a positive prognostic factor.

LD have often been characterized as including experienced control, enabling the dreamer to alter dream events (e.g., Gackenbach, 1988). Accordingly, LD has been related to (waking personality) internal locus of control (Blagrove and Tucker, 1994; Blagrove and Hartnell, 2000; Patrick and Durndell, 2004). Because control over dream events is considered an inherent part of LD, control items have often been included as indicators of lucidity (e.g., Watson, 2001) or mentioned as part of the LD definition (e.g., Tart, 1988). However, studies disentangling dream awareness from control have shown that uncontrolled LD are more common than controlled LD; this was found in a non-clinical sample of young adult undergraduate students (Aviram and Soffer-Dudek, 2018), a large sample of children and adolescents aged 6–19 (Voss et al., 2012), and a clinical sample of veterans suffering from post-traumatic stress disorder (PTSD) (Harb et al., 2016). Relatedly, in a preliminary study assessing whether LD may be used to practice a motor task, over half of lucid dreamers were unable to practice efficiently in the dream because of distractions, suggesting limited control. Interestingly, only those in control showed a performance benefit (Schädlich et al., 2017).

Additionally, even when there is control in LD, it is unclear whether this is necessarily beneficial for mental health. On one hand, veterans with PTSD whose nightmare distress decreased exhibited an increase in LD control (Harb et al., 2016). Also, students reporting high LD control reported less psychopathological symptoms than those reporting low LD control (Aviram and Soffer-Dudek, 2018). On the other hand, Mota et al. (2016) found, contrary to their hypothesis, that individuals suffering from psychotic symptoms had significantly higher LD control compared to healthy participants. They suggested that LD in a psychotic population is not recommended because they may further empower deliria and hallucinations, favoring internal over external reality. Indeed Holzinger (2014) suggested that some individuals may misuse LD, and caution should be exerted especially regarding psychotic clients. Notably, despite very different samples, two different studies showed that lucid dreamers were not better off psychologically (i.e., did not have lessened symptoms) compared to non-lucid dreamers (Mota et al., 2016; Aviram and Soffer-Dudek, 2018). In other words, in both studies, specific LD characteristics were related to psychopathology, but mere LD frequency was not. Dream characteristics related to lessened symptoms are not just control but also confidence of the lucidity and dream length, together labeled as LD intensity (Aviram and Soffer-Dudek, 2018). Importantly, however, those who were high in intensity were not different in psychopathological symptom scales compared to non-lucid dreamers. Their advantage was only compared to those who had LD awareness coupled with low control or intensity. Further research is needed to continue to examine whether the combination of high dream awareness with low dream control might be indicative of psychopathology. Such a notion would be compatible with the finding that veterans with PTSD demonstrated a LD profile characterized by high dream awareness and low control (Harb et al., 2016). In another study, Jones and Stumbrys (2014) expected sports students reporting LD to have higher mental health and perceive themselves as physically fit. However, they found no relation to mental health and an inverse relation to reported physical fitness.

Importantly, LD have been advocated as a therapeutic approach (lucid dream therapy, LDT; Gavie and Revonsuo, 2010), training individuals in induction techniques. This is usually aimed at chronic nightmare sufferers so that they can gain control over their nightmares by altering the ending of the dream scenario. Although there is some preliminary evidence in favor of LDT for nightmare treatment, it is inconsistent, the sample sizes are small, the effects are weak, and there is a need for more research (Macêdo et al., 2019). The mechanism of change is unclear as several participants improved without achieving LD (Zadra and Pihl, 1997; Spoormaker et al., 2003; Spoormaker and van den Bout, 2006). Possibly, the mere idea that they can gain control, rather than dream awareness *per se*, was responsible for the improvement. Notably, there is no evidence supporting LDT over other empirically based therapies (Lancee et al., 2010). Thus, it is not yet clear whether training people to achieve LD is worthwhile. Finally, LD has not shown any beneficial effect for PTSD symptoms (Spoormaker and van den Bout, 2006; Lancee et al., 2010; Harb et al., 2016).

Research on LD induction has mainly explored whether LD may be efficiently induced and whether it may carry psychological benefits. However, possible adverse consequences of LD induction have scarcely been investigated. Below, I will suggest two variables worthy of such consideration: sleep quality and psychological reality–fantasy boundaries.

Sufficient good sleep quality and sleep hygiene are crucial for mental and physical health (e.g., Benca et al., 1992; Kahn-Greene et al., 2007; Cappuccio et al., 2010). In addition to insufficient or poor sleep, unusual dreaming may also be considered as a form of sleep disruption, when arousal or vigilance permeate nocturnal consciousness (Soffer-Dudek, 2017). LD theoretically also represent arousal within sleep, but they do not show the robust relationships with distress shown by other unusual sleep experiences (Soffer-Dudek, 2017). However, that conclusion was based mostly on studies that assessed LD by averaging dream awareness with dream control into a single measure. Moreover, those studies did not separate spontaneous LD from deliberate induction. These facts may have weakened the relationships with distress and sleep problems.

LD is a hybrid sleep–wake state, with increased activity in frontal areas, which are usually suppressed during sleep (Voss et al., 2009; Dresler et al., 2012). This neurocognitive evidence is compatible with the phenomenological evidence, i.e., LD are characterized by increased metacognition, insight, critical thinking, and vigilance/monitoring compared to normal dreaming. Indeed, a tendency for having LD was associated with higher neural activation in areas considered responsible for thought monitoring (Filevich et al., 2015). Although we generally regard critical thinking and meta-cognition as adaptive, they are not part of normal sleep and dreaming; our brain probably tends to inhibit prefrontal cortical activity in sleep for a reason. Thus, a question arises: is it possible that frequent engagement in LD (as may occur following deliberate attempts at induction) may disrupt sleep, possibly resulting in adverse effects for our health? This is a question worth exploring as there are correlational data showing a relationship between LD and sleep problems, poor sleep quality, and nightmares. Specifically, Schadow et al. (2018) found that LD were related to poor sleep quality in two samples: university students (*N* = 444) and a community sample (*N* = 1,380). Notably, the relation was higher in the latter, suggesting that perhaps student samples have limited variance in terms of sleep quality. The shared variance between LD and poor sleep was also shared with nightmares (as demonstrated by mediation analysis), raising several causal hypotheses: each of the three variables may be the origin of the others. Similarly, LD were associated with nocturnal awakenings (Smith and Blagrove, 2015). Despite these correlations, it may be claimed that LD is unlikely to disrupt sleep as most lucid dreamers do not spend much of their sleep-time in LD in absolute terms. Possibly, LD *induction* may be the culprit disturbing sleep; for example, the “wake back to bed” technique (LaBerge, 1985) requires deliberate sleep interruption. Indeed Smith and Blagrove (2015) showed that the use of the alarm clock “snooze” button significantly associated with LD, perhaps with LD resulting from those brief morning awakenings. In Aviram and Soffer-Dudek (2018), the frequency of attempting to deliberately induce LD using induction techniques (rather than spontaneous LD) was the factor associated with sleep problems, stress, dissociation, schizotypy, depression, and obsessive–compulsive symptoms. Relatedly, in a study where the experimental group underwent a LD intervention promoting LD induction and then followed with daily diaries, there was a robust correlation between LD and depression (Taitz, 2011).

The disruption of the sleep–wake cycle is inherently linked with indistinct boundaries between waking and sleeping conscious experience; sleepiness may permeate the waking state and arousal may pervade dreaming (Soffer-Dudek, 2017). This may be particularly true for LD induction as techniques such as reality testing (Levitan and LaBerge, 1989) or the reflection technique (Tholey, 1988) require, to some extent, disruption of sleep hygiene by deliberate confusion of the sleep and waking states (e.g., scheduled awakenings in the middle of the night or asking oneself “am I dreaming?” during the day). The blurring of boundaries between reality and dreaming are theoretically related to psychosis-proneness/schizotypy and dissociative symptoms. Indeed lucid dreamers had impaired reality monitoring, with more confabulatory errors (Corlett et al., 2014), and as mentioned, lucid control was heightened in a psychotic group (Mota et al., 2016). LD were suggested to be part of a continuum pertaining to bizarre cognitions during the day and during the night due to their correlation with dissociation, schizotypy, and unusual sleep experiences (Watson, 2001). Indeed they were associated with parapsychological experiences such as out-of-body experiences and apparitions (Alvarado and Zingrone, 2007). In a longitudinal exploration, Aviram and Soffer-Dudek (2018) demonstrated that those who reported engaging in deliberate LD induction had an increase in schizotypy and dissociative symptoms over the span of the following 2 months. This directional finding, predicting change over time, is superior to cross-sectional designs; however, experimental research exploring possible harmful effects of induction is needed.

In conclusion, it seems that we may be cultivating a shared blind spot by focusing solely on the possible beneficial effects of LD induction, without taking into account possible risks. Might frequent induction be deleterious to sleep hygiene and sleep–wake psychological boundaries? If so, is it worth it? And do potential risks pertain specifically to vulnerable individuals, e.g., those high in baseline dissociation/schizotypy? We need more research to answer these questions with confidence. I hope LD researchers will consider these questions in their future research.

## Author Contributions

The author confirms being the sole contributor of this work and has approved it for publication.

### Conflict of Interest

The author declares that the research was conducted in the absence of any commercial or financial relationships that could be construed as a potential conflict of interest.

## Footnotes

^1^“Lucid Dreaming—Dream Views” Forum (n.d.). Available online at: https://www.dreamviews.com/ (accessed October 2, 2019).
